# Inspiratory muscle training in children and adolescents living with neuromuscular diseases: A pre-experimental study

**DOI:** 10.4102/sajp.v77i1.1577

**Published:** 2021-08-31

**Authors:** Anri Human, Brenda M. Morrow

**Affiliations:** 1 Department of Physiotherapy, Faculty of Healthcare Sciences, Sefako Makgatho Health Sciences University, Garankuwa, Pretoria, South Africa; 2 Department of Health and Rehabilitation Sciences, Faculty of Health Sciences, University of Cape Town, Observatory, South Africa; 3 Department of Paediatrics and Child Health, Red Cross War Memorial Children’s Hospital, University of Cape Town, Rondebosch, South Africa

**Keywords:** neuromuscular disease, pulmonary function, inspiratory muscle strength, inspiratory muscle training, children

## Abstract

**Background:**

Children with neuromuscular diseases (NMD) are at risk of morbidity and mortality because of progressive respiratory muscle weakness and ineffective cough. Inspiratory muscle training (IMT) aims to preserve or improve respiratory muscle strength, thereby reducing morbidity and improving health-related quality of life (HRQoL).

**Objectives:**

To describe the safety and feasibility of a 6-week IMT programme using an electronic threshold device (Powerbreathe®). Any adverse events and changes in functional ability, spirometry, peak expiratory cough flow (PECF), inspiratory muscle strength and HRQoL (Pediatric Quality of Life [PedsQL]) were recorded.

**Methods:**

A convenience sample of eight participants (*n* = 4 boys; median [interquartile range {IQR}] age: 12.21 [9.63–16.05] years) with various NMD were included in a pre-experimental, observational pre-test post-test feasibility study. Training consisted of 30 breaths, twice daily, 5 days a week, for 6 weeks.

**Results:**

There were significant pre- to post-intervention improvements in upper limb function and coordination (*p* = 0.03) and inspiratory muscle strength: maximum inspiratory mouth pressure (Pimax) (*p* = 0.01); strength-index (*p* = 0.02); peak inspiratory flow (PIF) (*p* = 0.02), with no evidence of change in spirometry, PECF or HRQoL. No adverse events occurred and participant satisfaction and adherence levels were high.

**Conclusion:**

Inspiratory muscle training (at an intensity of 30% Pimax) appears safe, feasible and acceptable, in a small sample of children and adolescents with NMD and was associated with improved inspiratory muscle strength, PIF and upper limb function and coordination.

**Clinical implications:**

Larger, longer-term randomised controlled trials are warranted to confirm the safety and efficacy of IMT as an adjunct respiratory management strategy in children with NMD.

## Introduction

Children with neuromuscular diseases (NMD) often present with respiratory morbidity because of underlying progressive respiratory muscle weakness (Chatwin et al. 2018; Mayer et al. 2017; Park et al. 2010). As the disease progresses in childhood and adolescence, lung volumes such as forced vital capacity (FVC) and peak expiratory flow (PEF) also reduce, increasing the risk for pulmonary complications (Birnkrant et al. 2018; Meier et al. 2017). The combination of poor cough, decreased airway clearance, restrictive lung disease and recurrent respiratory infections therefore leads to a progressive decline in pulmonary function and ultimately, respiratory failure (Boentert, Wenninger & Sansone 2017; Farrero et al. 2013; Mayer et al. 2017; Toussaint et al. 2018).

Inspiratory muscle weakness leads to limited sigh capacity, shortening and atrophy of respiratory muscles and chest wall, with associated poor ventilation, ineffective cough and decreased cardiorespiratory function (Benditt 2006; Kinane et al. 2017; Nicot et al. 2006). Similar to skeletal muscles, inspiratory muscles such as the diaphragm can be trained to improve force and endurance. Therefore, it is hypothesised that inspiratory muscle training (IMT) could ameliorate the decline of inspiratory muscle strength, thereby preserving pulmonary function and cough ability for longer and delaying the onset of respiratory muscle failure (Aboussouan 2009; Chatwin et al. 2003; Yeldan, Gurses & Yuksel 2008).

Variable reports on the effectiveness of IMT have been published, with outcomes potentially influenced by learning effect; the type, nature and severity of NMD; as well as the specificity, dosage and intensity of training (Gozal & Thiriet 1999; Koessler et al. 2001; Topin et al. 2002; Wanke et al. 1994; Winkler et al. 2000; Yeldan et al. 2008). A systematic review of IMT, limited to children and adolescents with NMD, reported that although IMT might be beneficial for improving inspiratory muscle strength and/or endurance, there was no published, scientifically rigorous research investigating its effect on morbidity (e.g. hospitalisation rate, respiratory infection frequency, adverse events) and health-related quality of life (HRQoL) (Human et al. 2017). These findings were supported by a recent systematic review, which evaluated the use of both inspiratory and expiratory muscle training in children and adults with NMD (Silva et al. 2019).

Despite the possible advantages, IMT in NMD remains controversial. The reason for this is that the use of IMT is mostly supported by low level evidence and there is a concern about its safety, particularly in patients with dystrophinopathies because of an associated increased risk of muscle damage, overexertion and fatigue during exercise (Eagle 2002; Finder et al. 2004; Guglieri & Bushby 2011; Koessler et al. 2001; Sander et al. 2000; Tidball & Wehling-Henricks 2014; Wanke et al. 1994). As a result of the aforementioned controversy regarding safety, contradictory and limited evidence supporting the use of IMT in children with NMD (Aboussouan 2009; Eagle 2002; Finder et al. 2004; Hull et al. 2012; Human et al. 2017), further research is warranted.

Our pre-experimental, observational study aimed to determine the safety, adherence to and acceptability of a 6-week IMT programme using an electronic threshold device (Powerbreathe®). Changes in functional ability, spirometry, peak expiratory cough flow (PECF), inspiratory muscle strength, adverse events and HRQoL (Pediatric Quality of Life [PedsQL]) were recorded.

## Methods

This was a pre-experimental, observational pre-test post-test study conducted in South Africa (SA) to determine the safety and feasibility of implementing IMT in children with NMD. In SA, specialised NMD centres are limited and therefore a non-probability convenience, purposive sampling frame was used. The two sites for recruitment were schools in Pretoria (Gauteng) that cater for a variety of children with special needs (physical and cognitive) and had full-time physiotherapists available to monitor IMT on a daily basis.

A convenience sample of nine children (5–18 years), with a confirmed diagnosis of NMD, was initially identified (from February to June 2017) from two schools catering for children with special needs. Children were excluded from participating if they were terminally ill, had a vital capacity (VC) < 25% predicted, or were enrolled in another clinical trial. One child with VC < 25% predicted was excluded after enrolment and eight children completed our study.

### Data collection tools

The first author performed a baseline bio-demographic assessment using routinely collected data extracted from patients’ files and measured weight and height. As a result of the majority of the participants being non-ambulant and four presenting with scoliosis, alternative measures for height measurement were implemented (left ulna length) in order to calculate height for spirometry test interpretation.

The level of ambulation was recorded and the functional ability of the lower and upper limbs was assessed. Lower limb function was measured subjectively using the 10-point Vignos scale, where 10 is the lowest possible score (confined to a bed) and one the highest (walks and climbs stairs without assistance). All participants were cognitively able to identify an appropriate Vignos score. Upper limb function was assessed objectively with the Brooke Scale, a six-point scale, where six is the lowest value (cannot raise hands to the mouth and have no functional hand movement) and one the highest (child can abduct their arms above their head in a full circle) (Lue et al. 2009).

Ten selected items of the Motor Function Measure (MFM) (items 14–23), with participants in a seated position (either in a chair or their wheelchair), were used to determine upper limb function and coordination. These items were selected so that all participants could be tested in a similar manner, despite their level of ambulation and could be performed in approximately 10 min (Bushby & Connor 2011; Mazzone et al. 2012). Each item on the MFM is scored on a four-point scale, with zero if the participant is unable to perform the task or cannot maintain the starting position whilst performing the task, and three reflects the ability to perform the task with a controlled (normal) movement pattern (Iwabe et al. 2014; MFM user manual: https://mfm-nmd.org/obtenir-un-manuel-utilisateur/https://www.statisticshowto.datasciencecentral.com/cohens-d/). Therefore, in this study, participants’ MFM score was expressed out of 30, for the 10 items.

**FIGURE 1:**
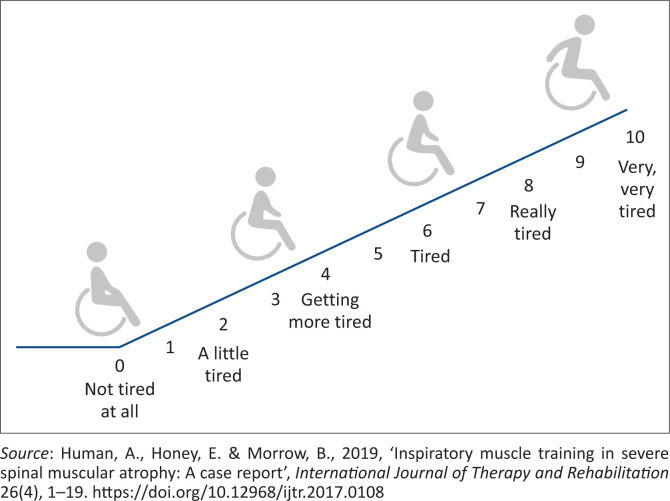
The OMNI scale used for participants to describe their level of perceived exertion.

Pulmonary function, safety, adherence, acceptability and HRQoL were assessed as follows:

1. Relaxed and forced spirometry:
  Slow vital capacity (VC), FVC, forced expiratory volume in one second (FEV_1_) and PEF were measured pre- and post-intervention, with participants in a sitting position and using a portable spirometer (MicroLoop ^TM^ Spirometer, Carefusion, Germany).
  At least three successful spirometry, cough ability (PECF) and inspiratory muscle strength (Pimax; sniff nasal inspiratory pressure [SNIP]) attempts were recorded, but testing at times continued up to five repetitions (Caruso et al. 2015; Farrero et al. 2013; Kinane et al. 2017; Meier et al. 2017; Miller et al. 2005; Nicot et al. 2006). The best of three satisfactory efforts, with acceptable variation < 20%, was used for analysis (Benditt 2006; Fauroux & Aubertin 2007; Stefanutti et al. 2000).
  In addition, the FVC and FEV_1_ z-scores were calculated (Global Lung Function Initiative [GLI]): http://gligastransfer.org.au/calcs/spiro.html) by the first author.
2. Cough ability:
  Unassisted PECF was assessed pre- and post-intervention using a Mini-Wright Peak Flow Meter (Clement Clarke International Ltd, UK) (Chiang, Mehta & Amin 2018; Park et al. 2010).
3. Inspiratory muscle strength:
  Inspiratory muscle strength (Pimax) was measured every 2 weeks in order to inform titration of IMT intensity.
  Inspiratory mouth pressure (Pimax) (from residual volume [RV]) and SNIP (from functional residual capacity [FRC]) were measured, in a sitting position, using an electronic handheld mouth pressure meter (MicroRPM^TM^; Carefusion, United Kingdom) (Chiang et al. 2018; Fauroux & Aubertin 2007; Hull et al. 2012; Park et al. 2010; Stefanutti et al. 2000; Yeldan et al. 2008). Sniff nasal inspiratory pressure was measured by occluding the left or right nostril (depending on the participant’s preference) with a nasal probe that ensured complete nasal passage closure whilst the other nostril remained open. The same size probe was used with each follow-up assessment every 2 weeks. The best of three attempts of maximum inspiratory effort was recorded.
  Peak inspiratory flow (PIF) (L/s) and strength (S)-index (cmH_2_O), with performance of one maximum inhalation from RV, were measured with the Powerbreathe K3® inspiratory threshold training device (HaB International Ltd, United Kingdom).
4. *Safety*:
  Any adverse events related to IMT were documented daily. Adverse events were classified as mild or moderate, which could include nausea or vomiting; pain or discomfort and gastric distension; whilst severe adverse events could include bradycardia, desaturation and/or hypoxia or barotrauma such as a pneumothorax. To monitor the participants’ perceived exertion during assessment and IMT (before and after training), a visual adapted 10-point Borg scale (OMNI scale) was used (Figure 1).
5. Adherence:
  The number of completed IMT training episodes over the study period was downloaded from the threshold IMT devices (Powerbreathe K3®) and noted in the IMT adherence diaries.
6. Acceptability:
  Satisfaction with the IMT intervention was rated on a 10-point visual analog scale (VAS) and the experience of IMT as verbally reported by participants and physiotherapists at the school was noted as open-text responses, after the 6-week intervention period.
7. Health related quality of life:
  Self-reported, age-appropriate PedsQL Inventory^TM^ (PedsQL Generic Score Scale^TM^) questionnaires, for participants were completed pre- and post-intervention, with assistance from the first author as needed. The questionnaire consists of four subdomains: physical, social, emotional and school domain. The total PedsQL score and the Physical and Psychosocial (combined score of emotional, social and school functioning) domains were documented (Iannaccone et al. 2009; Varni, Seid & Kurtin 2001).

### Inspiratory muscle training protocol

Inspiratory muscle training was performed for 6 weeks with an electronic handheld tapered-flow threshold device (Powerbreathe K3 ®, HaB International Ltd, United Kingdom). This device provides a visual stimulus for training, as every breath is counted down from 30, provided the breath quality is adequate to reach the preset threshold value in order to open the valve.

Participants performed IMT twice a day, 5 days a week, unless the child was absent from school or could not train because of other reasons. Participants started with three sets of 10 breaths (3 × 10 breaths), with a rest-interval of less than 60 s in-between sets, twice a day and progressed until they were able to complete 30 breaths consecutively without resting. The physiotherapists at the respective schools monitored the IMT sessions daily. Intensity for training was set at 30% of the participant’s best Pimax value, as measured at baseline and every 2 weeks by one of the authors and a research assistant. The intensity level was based on both evidence and the manufacturer guidelines suggesting that this is the minimum intensity required for improved inspiratory muscle strength and/or endurance (Hill et al. 2010; Lӧtters et al. 2002).

### Data management and analysis

Data were tested for normality using the Shapiro–Wilks *W* test and the relevant parametric (e.g. *t*-test for dependent samples) or non-parametric descriptive and inferential statistical tests were applied (Statistica v13; StasSoft Inc, Tulsa, United States of America). The significance level was set at *p* < 0.05.

Cohen’s *d* was calculated to estimate effect sizes for change in PedsQL scores (https://www.statisticshowto.datasciencecentral.com/cohens-d/).

### Ethical considerations

This study was designed as a pilot study of a registered clinical trial (PACTR201506001171421). Approval was obtained from the institutional Human Research Ethics Committee (513/2015), school boards and principals, as well as Department of Education (Gauteng). Informed consent was obtained from parents or legal guardians of all participants and assent was obtained from the child participants.

## Results

### Bio-demographic description and functional ability of participants

Eight IMT-naïve, non-ventilated children (four boys and four girls; median [interquartile range {IQR}] age 12.21 [9.63–16.05] years), with a variety of NMD (Duchenne muscular dystrophy [DMD] [*n* = 3], spinal muscular atrophy [SMA] [*n* = 3]; myopathy [*n* = 1] and degenerative neuropathy [*n* = 1]) participated in our study from February to June 2017. Two patients presented with cardiopulmonary comorbidity, including right ventricular hypertrophy and pulmonary hypertension; whilst one patient had a confirmed diagnosis of attention deficit hyperactivity disorder (ADHD) and mild cognitive impairment, but was still able to follow instructions. Four presented with spinal deformities, of which three had a thoracic scoliosis and one a thoracic kyphosis. Three participants had undergone previous surgical procedures including scoliosis repair, bilateral hip surgery for correction of dislocation and muscle lengthening/tendon release procedures.

The mean (± standard deviation [SD]) weight for age (WFA) z-score was -2.64 (± 2.66) and Body mass index (BMI) of -7.92 (± 7.53), suggesting severe wasting in most cases. For level of mobility, most participants (*n* = 6) were non-ambulant and were using a wheelchair (Table 1).

**TABLE 1:** Baseline bio-demographic data and functional ability of participants (*n* = 8).

Participant	Gender	Age (years)	NMD type	Weight for age (z-scores)	BMI (z-scores)	Mobility	Baseline (Brooke scale)	Baseline (Vignos scale)
1	Female	17.58	Myopathy	−3.31	−4.61	PA	2	7
2	Male	11.25	DMD	0.51	−0.42	A	1	2
3	Female	9.83	SMA	−5.62	−22.86	NA	3	9
4	Female	9.42	Neuropathy	1.06	0.04	NA	3	9
5	Male	15.42	DMD	−5.95	−14.28	NA	3	9
6	Male	16.67	DMD	−3.64	−7.51	NA	3	9
7	Female	13.17	SMA	−3.49	−7.29	NA	3	9
8	Male	8.33	SMA	−0.66	−6.42	NA	3	9

**NMD, Neuromuscular disease; BMI, Body mass index; PA, partially ambulant; DMD, Duchenne muscular dystrophy; A, Ambulant; SMA, spinal muscular atrophy; NA, Non-ambulant.**

Regarding functional ability, the median for upper limb function was three on the Brooke scale (ability to raise a glass of 180 mL water to the mouth, but unable to raise hands above their heads) and nine on the Vignos scale for the lower limbs (using a wheelchair). Both the Brooke (*p* = 0.11) and Vignos (*p* > 0.99) scales did not show any statistically significant change between baseline and 6 weeks (post-intervention), based on the Wilcoxon matched pairs test (Table 2). However, upper limb function and coordination (MFM) improved significantly from pre- to post-intervention (*p* = 0.03) (Table 2).

**TABLE 2:** Pre-post inspiratory muscle training variables.

Variable	Baseline		6 weeks (post-intervention)		*p*
	Central tendency	Dispersion measure	Central tendency	Dispersion measure	
VC (L)	1.44	0.66	1.56	0.83	0.18
FEV_1_ (L)	1.37	0.56	1.37	0.65	0.99
FEV_1_ z-scores	−3.84	0.87	−3.94	0.96	0.62
FVC (L)	1.54	0.62	1.55	0.62	0.98
FVC z-scores	−4.31	1.26	−4.33	1.07	0.94
PEF (L/min)†	150.5	98.5–168	161	121–196	0.05‡
FEV_1_/FVC (%)	93.38	5.50	92.00	5.55	0.65
Pimax (cmH_2_O)	37.88	14.60	48.13	16.45	0.01
Pimax % predicted	60.66	23.02	76.38	24.70	0.01†
SNIP (cmH_2_O)	37.13	20.68	40.38	20.07	0.60
PIF (L/s)	1.20	0.60	2.24	0.98	0.02
S-index (cmH_2_O)	23.88	9.88	41.88	16.15	0.02
PECF (L/min)	198.13	100.43	214.38	102.42	0.63
Brooke scale (/6)†	3.00	3.50–3.00	2.5	1.50–3.00	0.11‡
Vignos scale (/10)†	9.00	8.00–9.00	9.00	8.00–9.00	1.00‡
MFM (/30)	25.75	1.83	26.75	1.39	0.03
Child PedsQL total (%)	64.99	16.04	73.56	16.97	0.15
PedsQL (physical domain) (%)	65.23	30.45	65.50	23.0	0.97
PedsQL (psychosocial) (%)	64.80	12.83	77.08	17.10	0.10

**VC, vital capacity; FEV, forced expiratory volume; PEF, peak expiratory flow; FVC, forced vital capacity; Pimax, m**aximum **inspiratory pressure; SNIP, sniff nasal inspiratory pressure; PIF, peak inspiratory flow; PECF, peak expiratory cough flow; MFM, Motor Function Measure; PedsQL, Pediatric Quality of Life.**

All continuous variables are mean (± SD), unless otherwise stated;

† , Median (IQR);

‡ , Wilcoxon.

Participants were also asked if they followed any specific home programme. Two indicated that they were performing range of motion (ROM) exercises, which included passive movements and stretches daily or at least twice a week. Half of the participants performed some form of breathing exercise at least once a day. Most used manually assisted cough (MAC) techniques only during acute infections. None of the participants used mechanical insufflation-exsufflation (MI-E), lung volume recruitment (LVR) techniques, or other cough augmentation techniques such as glossopharyngeal breathing (GPB) or breath-stacking with a resuscitation bag or ventilator, which is similar to a previous SA survey (Human, Corten & Morrow 2021).

### Pulmonary function

#### Spirometry and cough ability

All participants had a restrictive pulmonary pattern of varying degrees, which remained unchanged pre- and post-intervention. There was also no change in spirometry values or cough ability (PECF) from baseline to 6 weeks post intervention (Table 2).

#### Inspiratory muscle strength (Maximum inspiratory pressure, sniff nasal inspiratory pressure, peak inspiratory flow and strength-index)

**FIGURE 2:**
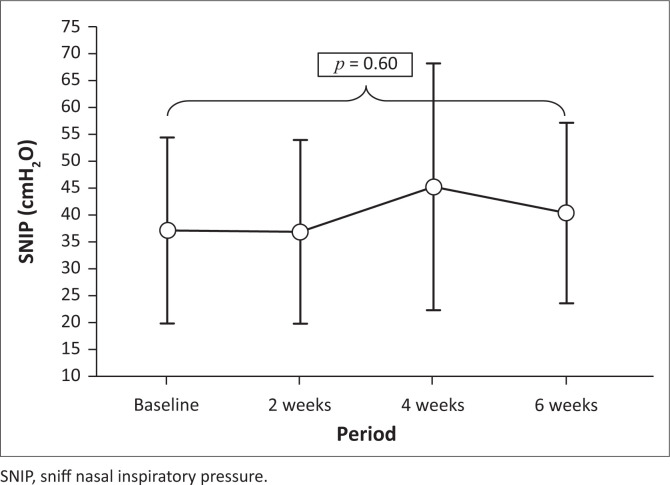
Mean sniff nasal inspiratory pressure change over training period (ANOVA, *p* = 0.39).

Measures of inspiratory muscle strength of Pimax (*p* = 0.01), PIF (*p* = 0.02) and S-index (*p* = 0.02) improved significantly from baseline (Table 2), whilst SNIP did not show any significant change post-intervention (*p* = 0.60) (Table 2), or at any other point over the intervention period (Figure 2; analysis of variance [ANOVA] *p* = 0.39).

**FIGURE 3:**
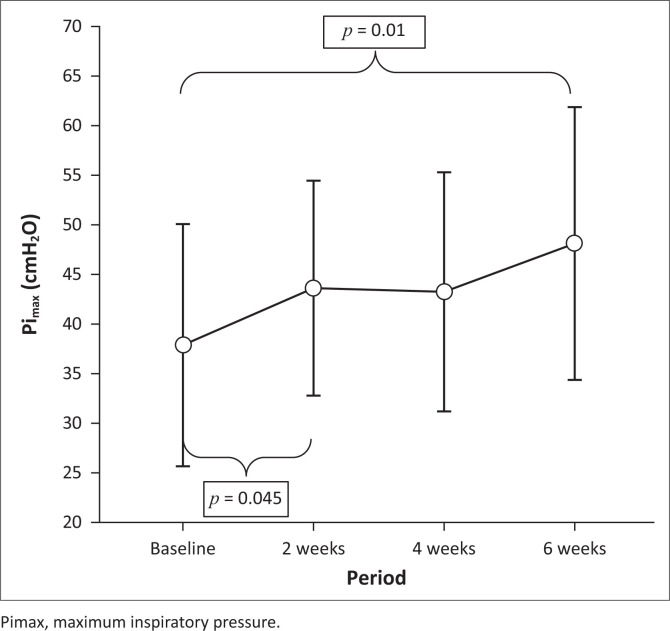
Mean maximum inspiratory pressure change over training period (ANOVA, *p* = 0.02).

**FIGURE 4:**
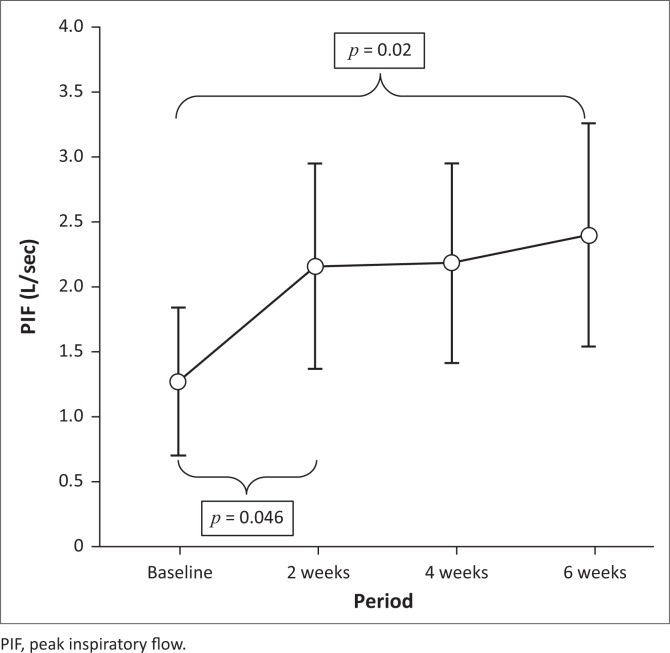
Mean peak inspiratory flow change over training period (ANOVA, *p* = 0.007).

Maximum inspiratory mouth pressure (Pimax) and the ability to create airflow when taking a deep breath (PIF) increased significantly throughout the intervention period and from pre- to post-test (Table 2; Figure 3 & 4).

On *post hoc* analysis, the greatest increase in both Pimax (*p* = 0.045) and PIF (*p* = 0.046) occurred during the first 2 weeks of intervention (Figure 3 & 4).

We did not find any association between any of the bio-demographic data (age, sex, height and BMI) or any pulmonary function measure (spirometry, PECF and inspiratory muscle strength) and no significant association between sex and change in Pimax (ΔPimax).

#### Inspiratory muscle training protocol and adherence

The mean (± SD) baseline IMT intensity was 11.4 (± 4.5) cmH_2_O, increasing to 13.6 (± 3.9) cmH_2_O and 13.4 (± 4.4) cmH_2_O at 2 and 4 weeks, respectively. Similar to Pimax, S-index and PIF; a significant change was observed over the period of IMT (*p* = 0.035), especially during the first 2 weeks (*p* = 0.020). Adherence diaries were used by the physiotherapists at the schools to monitor each participant’s training session and to document levels of exertion and any adverse events. Participants completed 51 (47–53) median (IQR) training sessions recorded by the Powerbreathe® device (out of a total possible 60 sessions) during the intervention period.

#### Adverse events and safety of inspiratory muscle training

No adverse events directly attributable to IMT were reported during the 6-week intervention period and on average, over the first 2 weeks of IMT, the majority of participants reported a low OMNI score, which indicates low levels of perceived exertion.

#### Health-related quality of life and acceptability of inspiratory muscle training

Although there was a slight improvement in HRQoL (total PedsQL [%]) as reported by the children from baseline to post-intervention, this was not statistically significant (*p* = 0.15) (Table 2). The percentage change in the physical and psychosocial domains (social, emotional and school performance) were both positive with a mean (± SD) of 0.28 (± 17.49) and 12.29 (±18.36), respectively, but neither were significant (*p* = 0.97; *p* = 0.10) (Table 2). When calculating the Cohen’s *d* for effect sizes, the Child PedsQL total score showed a medium effect size (0.52) and the psychosocial domain a large effect size (0.81).

Based on subjective feedback post-intervention, overall satisfaction with the IMT programme, on a 10-point visual analog scale, was extremely high, with a median (IQR) of 9 (9–10). All participants (*n* = 8) and physiotherapists involved in our study agreed that, if provided the opportunity, they would prefer to continue with IMT as part of their management programme. Open-text responses relating to the acceptability of IMT are provided in Table 3.

**TABLE 3:** Open-text responses from participants and physiotherapists (*n* = 8).

What did the participants have to say about IMT?	What did the physiotherapists have to say about IMT?
It is nice and relaxing!	Improvement noticed in his posture, breathing pattern and endurance.
It helps me with breathing, I am less out of breath and my lungs work better.	Improved posture, confidence he even corrected others who participated in the study. He also presented with improved endurance and academic performance.
My lungs are better, I breathe better (more normal) and I can sing louder in the choir!	Improved posture, confidence and increase in voice volume/projection, as she speaks and sings louder. Decreased use of asthma medication (cortisone) since she started with IMT. Regarding morbidity, she presented with a decreased frequency of respiratory illness during the winter period as compared with previous years: only one upper respiratory tract infection and one case of gastro-enteritis during the 6-week training period.
Liked the training, it was nice.	When he started training, he was ill and had difficulty with IMT, after he received medication, he was much better and could start with training again. He did not fall ill again after he started training and his respiratory morbidity during the wintertime decreased (frequency of respiratory illness).
Helps me to improve and keeps me healthier. I can go shopping with my mother and come to the physiotherapy department without having to stop every now and again because I am short of breath.	Inspiratory muscle training will be more beneficial in some patients as compared with others. For this patient specifically, it was clinically relevant, and she was very dedicated and will continue with training (patient adherence has a positive influence on the outcome of the intervention).
It was fun, and now I can run faster and longer!	Similar to the previous participant: certain patients might benefit more from IMT than others, depending on their diagnosis, clinical presentation and motivation. This patient was very dedicated, motivated to exercise and seems to benefit from the intervention (clinical presentation).
It makes my lungs better.	Any effort of maintenance is valuable for these patients and making them aware of effective breathing is to their advantage/provides clinical benefit for them.
Liked the training, it helps me with my breathing.	Any effort of maintenance is valuable for these patients and making them aware of effective breathing is to their advantage/provides clinical benefit for them.

IMT, inspiratory muscle training.

## Discussion

This is the first SA study to investigate the potential utility of IMT in children with NMD. This was a short-duration, pre-experimental, feasibility study with a small, heterogeneous convenience sample from one region, limiting internal and external validity. Despite these limitations, the results support the use of IMT in children with NMD with no adverse events, high participant adherence and satisfaction. Evidence of positive outcomes for pulmonary function (in particular Pimax, S-index and PIF) and suggested improvements in functional ability and HRQoL were found, similar to other studies (Human et al. 2019; McCool & Tzelepis 1995; Wanke et al. 1994; Winkler et al. 2000).

The clinical and bio-demographic profile of this cohort was similar to previous reports with the majority already non-ambulant, as expected for patients with DMD and SMA in this age range (median age of 12.2) (Gozal & Thiriet 1999; Meier et al. 2017; Nicot et al. 2006; Topin et al. 2002; Wanke et al. 1994; Winkler et al. 2000). Despite most participants having reasonable oral control, the majority were severely underweight based on z-scores for WFA (median = -3.40) and BMI (median = -6.86). Similar BMI alterations in a study amongst children and adults (7–23 years) with congenital muscular dystrophy (CMD) and SMA were reported by Marques et al. (2014), as four of their participants younger than 20 years were underweight, whilst only one was overweight and one obese.

Participants presented with moderate upper limb function (median Brooke scale = 3/6), generally good upper limb function and coordination (mean MFM score = 26/30) and poor lower limb function (median Vignos 9/10) at baseline. These findings were similar to a study amongst children and adults (*n* = 179) with a variety of muscular dystrophies, where patients with DMD presented with a mean (± SD). Brooke score of 3.2 (± 1.9) and Vignos score of 7.1 (± 3.1) (Lue et al. 2009). As expected, there was no change in lower limb function following IMT in this feasibility study, given the short duration of IMT and the fact that most participants were already non-ambulant (Vignos = 9).

The baseline Brooke upper limb score of 3/6 suggests that participants were in a time of transition in their upper limb function, with progressive inability to reach overhead. This transition period coincides with pulmonary functional loss (Birnkrant et al. 2018; Mayer et al. 2017; Meier et al. 2017). There was no statistical improvement in upper limb function; however, this may be explained in part by the short study duration, as the Brooke scale is usually only responsive to change over a longer period of observation (Birnkrant et al. 2018).

Reduced upper limb function has been associated with progressive loss of pulmonary function particularly, FEV_1_ and FVC (Birnkrant et al. 2018; Mayer et al. 2017; Meier et al. 2017). It was therefore interesting, albeit unexpected, to note a significant improvement in MFM scores (*p* = 0.03) following the 6-week IMT programme. Although statistically significant, the clinical relevance of a change of one point on the MFM score is however unclear, especially considering the high baseline scores and small sample size, and therefore this finding should be interpreted with caution. It is possible that improvements in diaphragmatic strength, cognitive awareness of posture, chest mobility and breathing patterns with IMT may have translated into better proximal stability and hence, improved upper limb function, but this warrants further research.

As a result of their advanced disease progression and decreased diaphragmatic function, low spirometry measurements were expected in this population. The mean FVC values were however notably lower than a cohort study of children with DMD (7–15.5 years) (Aslan et al. 2013; Hull et al. 2012; Kinane et al. 2017; Yeldan et al. 2008; Winkler et al. 2000) and there was no change in lung volumes or flows post-IMT intervention. Spirometry tests, especially forced manoeuvres that are volitional and effort dependent, might be difficult to perform in the presence of severe restrictive lung disease and cognitive deficits and should be considered in future studies (Boentert et al. 2017; Chiang et al. 2018; Farrero et al. 2013; Guglieri & Bushby 2011; Nicot et al. 2006; Toussaint et al. 2018). The fact that lung volumes did not decline during our study period may reflect the short intervention duration. Long-term studies with larger sample sizes are needed to determine if IMT might be able to counterbalance the natural decline of pulmonary function in patients with NMD.

Cough ability (PECF) for participants at baseline was within normal range for children of 4–18 years (Bianchi & Baiardi 2008) and did not change significantly following intervention. Despite being within normal range, the mean PECF at baseline was < 270 L/min, the value recommended for the initiation of cough augmentation to assist with airway clearance in patients > 12 years of age (Farrero et al. 2013; Finder et al. 2004; Hull et al. 2012; Toussaint et al. 2018). Despite an improvement in inspiratory muscle strength, which would enable a patient to take a deeper breath and improve their cough ability, participants’ improved inspiratory muscle strength (Pimax and PIF) did not translate into improved PECF, which is similar to Aslan et al. (2013). This finding may relate to the small sample size and/or the short duration of the intervention.

The inspiratory muscle strength values (Pimax and SNIP) at baseline for participants were similar. A slight, non-significant, improvement in mean SNIP post-intervention was observed, whilst Pimax showed a significant improvement after IMT, similar to other studies (Gozal & Thiriet 1999; Koessler et al. 2001; Wanke et al. 1994). Both SNIP and Pimax values at baseline and post-IMT Pimax values were below normative for children in the same age range (Chiang et al. 2018; Fauroux & Aubertin 2007), but within expected ranges for children with NMD (Chatwin et al. 2003; Stefanutti et al. 2000).

When the oropharyngeal muscles are affected in people with NMD, alternative inspiratory muscle strength measures, such as SNIP, might need to be considered because of poor oral control, which can render Pimax testing inaccurate (Birnkrant et al. 2018; Boentert et al. 2017; Chiang et al. 2018; Farrero et al. 2013). The advantages of SNIP is that the technique is reliable, might be more natural and easier to perform and does not require practise or oral control (Fauroux & Aubertin 2007; Gozal 2000; Nicot et al. 2006), however our participants showed a subjective preference for Pimax testing. The majority presented with moderate oral control, but because Pimax and SNIP values are strongly correlated (Nicot et al. 2006; Stefanutti et al. 2000), and one could be used as a surrogate for the other to indicate inspiratory muscle strength, our findings were unexpected. The  SNIP manoeuvre is still effort dependent and submaximal efforts can occur in patients who are ill or breathless (Gozal 2000). Measures of Pimax and SNIP are therefore not necessarily interchangeable, and SNIP should be considered as an additional measurement to Pimax (Caruso et al. 2015; Stefanutti et al. 2000).

Conversely, it has also been reported that the diaphragm activation pattern in patients with NMD during a sniff manoeuvre is unknown (Stefanutti et al. 2000). The different patterns of muscle activation and type of effort between Pimax and SNIP suggest that these tests might represent different aspects of inspiratory muscle function, which needs to be considered when planning for future clinical trials. The Pimax measurements and IMT in our study were both performed at RV, implying that measurement and training were of the same motor unit. This could also explain why SNIP values did not improve significantly, as measurements for SNIP are taken at FRC (Fauroux & Aubertin 2007; Hill et al. 2010; Hull et al. 2012; Stefanutti et al. 2000; Verma et al. 2019). At RV, passive recoil pressure of the chest wall is present, which could provide an overestimation of the patient’s inspiratory muscle strength, whereas at FRC the chest recoil is zero, which might be a more specific indication of true inspiratory muscle strength (Fauroux & Aubertin 2007). This hypothesis however requires confirmation in further studies. Other reasons for variations in values of inspiratory muscle strength measures between different studies that should be considered are the equipment/devices used, characteristics of the participants, their level of motivation and the experience of the assessor (Verma et al. 2019).

We also found a significant improvement in the ability to create airflow (PIF) when taking a deep breath (*p* = 0.02), despite all participants presenting with restrictive lung disease of varying severity. Threshold load training, as was used here, provides both pressure and flow load as a training stimulus. This could explain the observed improvement in both inspiratory muscle strength and inspiratory flow. The marked increase in Pimax, S-index, PIF and training intensity (cmH_2_O) observed during the first 2 weeks of training could be attributed to a learning effect related to improved respiratory muscle action and coordination commonly observed with the use of threshold IMT devices (Eagle 2002; Gozal & Thiriet 1999; Winkler et al. 2000). The fact that Pimax continued to improve after the first 2 weeks of intervention, albeit at a slower rate, suggests a possible true inspiratory muscle strength increase. The value of IMT, independent of the learning effect, requires further longer-term investigations.

No adverse events directly attributable to IMT were reported, similar to other IMT studies in children and adults with NMD (Wanke et al. 1994), even in patients with advanced disease progression and severe respiratory muscle weakness (McCool & Tzelepis 1995; Wanke et al. 1994; Winkler et al. 2000). Based on measurements, feedback and observation during this feasibility study, 30% of Pimax appears to be a safe intensity for children with NMD to train at but this requires confirmation in larger, controlled trials.

To the best of our knowledge, this is the first published report of HRQoL (based on PedsQL) of children with NMD in SA. Although we showed an improvement in the child-reported PedsQL total scores, this was not statistically significant (*p* = 0.15), possibly because of the small sample size, short study duration and limitations in a child’s understanding of some of the more abstract questions. However, the medium and large effect sizes for the change in PedsQL total and Psychosocial domain scores suggest that the effects of IMT may translate into improved HRQoL. Mid- and long-term studies are therefore recommended to determine the impact of IMT on these patients’ HRQoL and validation of instruments in the SA context is needed.

Non-adherence might pose a challenge with IMT programmes, especially in children and adolescents (Eagle 2002; Topin et al. 2002; Wanke et al. 1994), but in this study most participants were very motivated throughout the 6-week intervention period and adhered well to the intervention programme. This could be attributed to the extremely high reported participant satisfaction with IMT (median: 9/10) and the positive attitude of the physiotherapists, parents and caregivers involved (Table 3). Further qualitative research is recommended to explore the experience of children and their caregivers regarding pulmonary rehabilitation, inclusive of IMT.

### Limitations of the study and research recommendations

Although our study suggests possible advantages of IMT in patients with NMD, this was a feasibility study with a small sample size, which included children of both sexes, with a variety of NMD and large age range (8.33–17.58) sampled from only one province in SA, and without a control group. The heterogeneity of the conditions included, the varying levels of physical and cognitive maturity, and the lack of a control group precludes conclusions being made as to the direct association between IMT and the reported outcomes. Factors such as sex, age, ethnicity and growth can influence absolute spirometry values (Birnkrant et al. 2018; Chiang et al. 2018; Meier et al. 2017) and therefore norm-adjusted percentage predicted values or z-scores for pulmonary function (FVC & FEV_1_) were calculated. Although other studies have indicated a difference in inspiratory muscle strength between boys and girls (Fauroux & Aubertin 2007; Kinane et al. 2017; Tomalak, Pogorzelski & Prusak 2002), we did not find any association between bio-demographic data and pulmonary function (spirometry, PECF and inspiratory muscle strength). This can however be attributed to the small sample and should be considered in future clinical trials.

Furthermore, the variation in home programme adherence including the performance of stretches, passive movements and breathing exercises on a regular basis, could be confounding factors, which might influence the outcomes of upper limb and pulmonary function. The possible cognitive involvement commonly observed in children with dystrophinopathies such as DMD (Guglieri & Bushby 2011; Morrow et al. 2019) could influence the outcome of IMT and should be considered in future research. Furthermore, longer-term studies (> 6 weeks of IMT) might be necessary to determine if IMT can slow down the regression of pulmonary function and respiratory muscle strength in children with NMD (Winkler et al. 2000). As seen from our pre-experimental study, although not statistically significant, there was an improvement in self-reported HRQoL. The use of HRQoL outcome measures, for both patients and their caregivers, is recommended for future research to determine the effect of long-term IMT.

The convenience sample selection may also have introduced bias.

As a result of these limitations, external validity is compromised and although the results indicated possible clinical benefit of IMT, these findings cannot be extrapolated to larger NMD samples. To determine if IMT can bring a true change in inspiratory muscle strength and be sufficient to counterbalance the natural decline of pulmonary function in patients with NMD, larger and longer-term randomised controlled trials are needed before recommendations for clinical practice can be made.

## Conclusion

This was a safety and feasibility study that showed short-term IMT intervention with a threshold tapered flow handheld device, set at an intensity of 30% Pimax, was associated with improved inspiratory muscle strength, PIF and upper limb function and coordination amongst participants. Despite the fact that child-reported HRQoL did not improve significantly following the intervention, the qualitative responses from participants and physiotherapists suggested that the effects of IMT may translate into both improved function and quality of life. This pre-experimental study provides preliminary data supporting the need for adequately powered clinical trials to confirm the safety, feasibility and efficacy of the use of IMT in children with NMD.
